# Time Trends in Atrial Fibrillation-Associated Stroke and Premorbid Anticoagulation: Population-Based Study and Systematic Review

**DOI:** 10.1161/STROKEAHA.118.022249

**Published:** 2018-11-29

**Authors:** Gabriel S.C. Yiin, Linxin Li, Yannick Bejot, Peter M. Rothwell

**Affiliations:** 1 From the Centre for Prevention of Stroke and Dementia, Nuffield Department of Clinical Neurosciences, University of Oxford, United Kingdom (G.S.C.Y., L.L., P.M.R.); 2 Dijon Stroke Registry, EA 7460 Pathophysiology and Epidemiology of Cerebro-Cardiovascular Diseases (PEC2), University Hospital of Dijon, University of Burgundy, France (Y.B.).

**Keywords:** atrial fibrillation, incidence, population, prospective studies, stroke

## Abstract

**Background and Purpose—:**

Prevalence of atrial fibrillation (AF) is increasing, but the impact on overall burden of stroke is uncertain, as is the proportion that could be attributed to under anticoagulation. We did a population-based study of AF-associated stroke and a systematic review of time trends in other stroke incidence studies and of rates of premorbid anticoagulation.

**Methods—:**

The proportion of incident strokes with associated AF was determined in the OXVASC (Oxford Vascular Study; 2002–2017) and in other prospective, population-based stroke incidence studies published before December 2017. Proportions were pooled by Mantel Haenszel methods, and the pooled percentage of cases with premorbid anticoagulation was determined. Analyses were stratified by the age of study population, mid-study year, country, and ethnicity.

**Results—:**

Of 1928 patients with incident ischemic stroke in OXVASC, 629 (32.6%; 95% CI, 30.5–34.7) were AF associated, consistent with the pooled estimate from 4 smaller studies over the same study period (608/1948; 31.2%, 30.0–32.4; *P*_het_=0.80). The pooled estimate from all studies reporting premorbid AF over 25 million person-years of observation (1960 onwards; 33 reports) was lower (18.6%, 16.8–20.3) and more heterogeneous (*P*_het_<0.0001), but 62% of heterogeneity was explained by the age of study population, study period, country, and ethnicity. The proportion of incident strokes on premorbid anticoagulation increased over time, both for ischemic stroke in OXVASC (2002–2007: 15.1%, 2008–2012: 19.6%, and 2013–2017: 35.9%; *P*_trend_<0.0001), and across all studies (*P*=0.002), but the pooled estimates suggested substantial undertreatment even in the most recent periods (2001–2015: 25.7%, 21.1–30.3 and ≥2010: 31.6%, 18.2–44.9).

**Conclusions—:**

About 1 in 3 incident ischemic strokes are still AF associated, due partly to low rates of anticoagulation for known prior AF, which therefore represents a major public health opportunity to reduce the burden of stroke.

## Abstract 2

Supplemental Digital Content is available in the text.

Atrial fibrillation (AF) is the most common sustained cardiac arrhythmia, one of the most common cardiovascular conditions overall^1^ and affects up to 1% to 2% of people worldwide, rising to over 10% at age >80.^2^ In high income countries, the number of individuals affected is projected to increase substantially over the next 4 decades (Figure I in the online-only Data Supplement). Parallel increases in low-to-middle income countries are expected because of the epidemiological transition and an overall rise in noncommunicable forms of heart disease are also likely.^2^ AF not only confers a 3- to 5-fold increased risk of stroke at all ages,^3^ but AF-associated strokes are also more severe, resulting in greater disability, institutionalization and healthcare cost.^4^

Prospective population-based studies remain the gold standard in assessing stroke incidence and outcome, the need for stroke-related prevention strategies and health services, geographic and secular trends in stroke burden, and for increasing public awareness and education.^5^ Criteria used to judge the quality of population-based studies were first published^6^ in 1987 and later updated over the years.^5,7^ In the few European population-based studies that fulfill the “ideal criteria” examining AF-associated stroke,^8–12^ the proportion of any AF ranges from 18% to 33% spanned >30 years. The underuse of oral anticoagulation in patients with AF has also been well-established across studies,^13,14^ but data on the burden of potentially preventable stroke are lacking at the population level.^8^

Although no population stroke incidence study has had detailed data on anticoagulation use in the underlying population, these studies can be used to measure the consequence of underuse of anticoagulation (ie, the number of ischemic strokes that occur in people with known prior AF who were not anticoagulated). Therefore, we aimed to determine the proportion of incident strokes that are AF associated, stratified by premorbid anticoagulation, in a large population-based stroke incidence study in Oxfordshire, United Kingdom, and in a systematic review of all other similar published studies.

## Methods

Requests for access to data from OXVASC (Oxford Vascular Study) will be considered by the corresponding author.

### Study Population

OXVASC is a population-based study of the incidence and outcome of all acute vascular events in a mixed urban/rural population of Oxfordshire, United Kingdom. Methods and definitions of events have been reported previously.^8,15^ Briefly, the study population comprises 92 728 individuals registered with 9 general practices (≈100 family doctors) that refer patients to the main Oxford Hospitals. Ascertainment of acute vascular events started in April 1, 2002, and is ongoing. Case ascertainment uses multiple overlapping methods of hot and cold pursuit and has been shown to be near complete.^15^ For this article, only incident ischemic strokes ascertained up to March 31, 2017, were included. OXVASC has local research ethics committee approval.

All patients gave informed consent to participate in OXVASC, or assent was gained from a relative. Patients were seen by study physicians as soon as possible after presentation. ECG done routinely at baseline as part of clinical care and regular ambulatory cardiac monitoring was completed after October 2010. Clinical study reports of all strokes were reviewed by the senior study neurologist. We obtained additional premorbid baseline clinical characteristics, lipid profile, BP measurements, and medications by interviewing patients and relatives and by review of primary care and hospital records.

Stroke was defined as an event with appropriate symptoms lasting longer than 24 hours.^15^ AF-associated ischemic stroke was defined as those associated with paroxysmal, persistent, or permanent AF^8^ (defined on the basis of an ECG showing either absent p waves or atrial flutter with an irregular ventricular response) documented before the event, at the time of assessment, or within 1 month after the event, including cases identified on short-term cardiac monitoring. Patients were subdivided according to whether AF had been documented before the acute event (prior AF), with confirmation from primary care or hospital records.

All patients had face-to-face follow-up at 1, 6, 12, 24, 60, and 120 months after the event to assess the outcome. For patients who had moved out of the study area, telephone follow-up was performed. Follow-up was conducted via a carer if the patient was unable to participate, for example, due to dementia.

### Systematic Review

We performed this systematic review according to the Preferred Reporting Items for Systematic Reviews and Meta-Analyses criteria.^16^ A literature search using PUBMED (1950–November 2017), Cochrane database (1972–November 2017), and EMBASE (1974–November 2017) for population-based studies of stroke was completed using search terms in Table I in the online-only Data Supplement. Reference lists of included studies and relevant systematic review^17^ were hand searched. There was no language restriction.

Studies were included if they met the “ideal criteria” for population-based stroke incidence studies:^17^ complete, population-based case ascertainment based on multiple overlapping sources of information; standard WHO definition of stroke; incident stroke cases reported; data collection over whole years; no upper age limit for population studied; and a prospective study design. They were also required to contain raw numbers for calculation of rates of AF or premorbid anticoagulation. We attempted to contact authors with time trend data if any raw numbers within the studies are unclear. We also included updated data from OXVASC. The abstracts of all articles identified from initial searches were reviewed by G.S.C. Yiin and L. Li and both authors reviewed the full text of all eligible studies. Where there was >1 publication on a cohort of patients, data on prior AF rate were taken from the most recent publication. Where data from later cohorts were added to those from earlier cohorts, from which data had already been published, the numbers in the combined cohort were used in the analysis. In cases of disagreement between authors about the eligibility of studies or data extraction, the consensus was reached through joint reassessment.

For each study, we recorded the study region, study period, population ethnicity, mean age with SD, year of publication, population size, observed person-years, total number of events, premorbid or total (premorbid and new) AF prevalence with the corresponding rate of premorbid anticoagulation, presence of AF definition, and ECG completion rate. When studies used incident stroke and incident ischemic stroke as denominators, we chose the denominator which was the most consistently used in sequential articles so as to compare the time trends of AF rates.

### Statistical Analysis

The proportion of incident strokes with associated AF was determined in the OXVASC (2002–2017) and related to baseline clinical characteristics and to study period (5 years bands). We used χ^2^ or Fisher exact test to compare categorical variables and Student *t* test for continuous variables, with statistical significance defined as *P*<0.05.

The main analysis was based on population-based studies reporting prevalence of prior or total AF within incident or incident ischemic strokes. We calculated the relative rate of AF with 95% CI for each study using the Mantel-Haenszel method and also allowing for extrabinomial variation because the standard methods of calculating 95% CIs produce artificially narrow intervals if there is heterogeneity of rates between different studies. We compared the pooled relative rates of AF according to the country of origin and study period of studies. The studies were later separated into those that used incident stroke and incident ischemic stroke respectively as denominators for subgroup analyses. Analyses of heterogeneity of prevalence across studies were done with χ^2^ tests.^18^ We used fixed effects analysis for pooled rates unless there was evidence of heterogeneity, in which case random effects analysis was used. If there were <2 studies in any stratified analysis, we added 0.1 to the 2 empty cells in the 2×2 table to enable graphic representation and CI estimation. If there were no patients with newly detected AF in any study, we added 0.1 to the numerator cell alone for the same reason.

The proportion of overall heterogeneity of the prevalence of prior AF across all studies that can be accounted for by the above subcategorisations was calculated by an inverse variance weighted linear regression of AF (percentage) against the study country, study period, population ethnicity, and mean age in univariate and multivariate analyses. We generated bubble plots to illustrate the time trends of all the pooled rates for AF and premorbid anticoagulation with the size of each bubble representing the size of the denominator in each study. We used the funnel plot to assess for publication bias by comparing new AF rate against the standard error of the new AF rate. We used in-house software to generate the forest plots and performed all statistical analysis and graphical presentation using SPSS software version 22 and Microsoft Excel 2013 for Windows.

## Results

Of 1928 patients in OXVASC, the mean age was 74.5 years (SD 13.5), 958 (49.7%) were female, and 629 (32.6%) were associated with any AF. Compared with the non-AF group, patients with AF-associated stroke had a significantly (*P*<0.05) higher prevalence of most vascular risk factors and usage of preventative medications except for smoking, diabetes mellitus, hypercholesterolemia, venous thromboembolism, and use of lipid-lowering agents (Table). Of the 425 patients with ischemic stroke and prior AF, 102 (24.0%) received premorbid anticoagulation, and the anticoagulation rate had increased over time (2002–2007: 15.1%, 2008–2012: 19.6%, and 2013–2017: 35.9%; *P*_trend_<0.0001) but remained low at 16.8% for age ≥80.

**Table.**
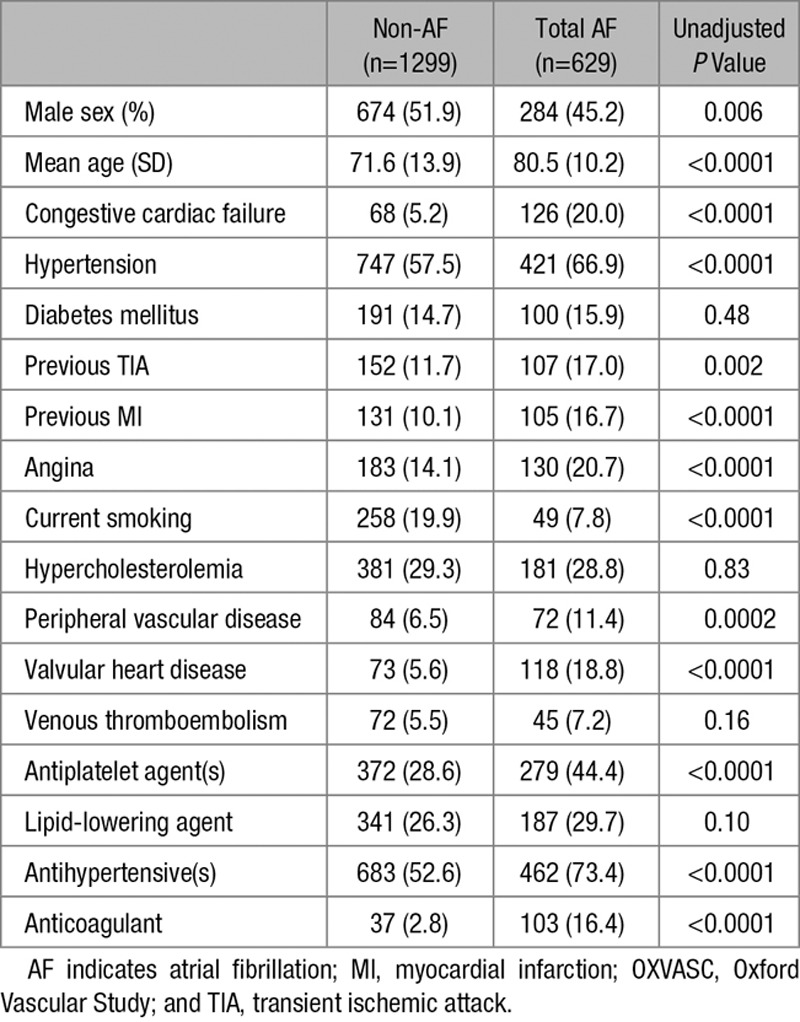
Baseline Characteristics of OXVASC Stroke Patients in Relation to the Prevalence of AF

The literature search identified 198 659 citations and 147 articles describing 57 population-based stroke incidence studies that met the eligibility criteria (Figure II in the online-only Data Supplement) for the systematic review.^8–12, 19–47^ We identified 33 articles reporting 31 studies from 22 countries and 38 study periods that contained data on prevalence of AF associated with incident strokes (Table IIA and IIB in the online-only Data Supplement).

**Figure 1.**
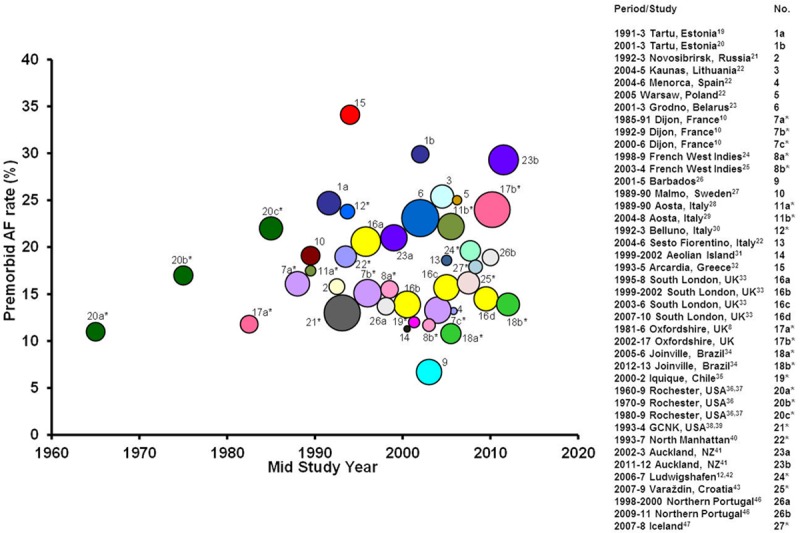
Premorbid atrial fibrillation (AF) prevalence in population-based studies of incident stroke or incident ischemic stroke.* Overall heterogeneity *P*<0.0001, of which 61.6% could be accounted for by the age of stroke population >70 y, mid-study year, country of origin and ethnicity (main determinant at 32.5%) of the population—Table III in the online-only Data Supplement.

Twenty-eight studies (including OXVASC) reported prevalence of premorbid AF among a total of 30 383 incident strokes from a combined study population of 10 809 215 people, with a total of about 25 million person-years of observation. There were 14 studies that reported data on all incident stroke and 14 that reported only incident ischemic stroke (Figure 1). The North Dublin^11,45^ and Udine studies^44^ only reported data on total AF and 5 additional studies (Dijon,^10^ Ludwigshafen,^12,42^, Iceland,^47^ Oxfordshire Community Stroke Project,^8^ and OXVASC) reported both prior and total AF. Of the 10 studies^8,10,19,20,24,25,28,29,33,34,36,37,41,46^ that had time trend data on prevalence of prior AF (Figure 1) all but 4^19,20,33,41,46^ used incident ischemic stroke as the denominator. Seven studies had a clear definition of AF^8–12,42^ and 7 studies reported the rate of ECG use.^8,11,12,29,35,41,43^ The Rochester study^36,37^ did not include atrial flutter in the AF group and only the North Dublin^11,45^ study reported the rate of echocardiogram and prolonged cardiac monitoring.

The pooled rate of prior AF among all incident stroke was 18.6% (95% CI, 16.8–20.3), but there was substantial heterogeneity between studies (*P*_het_<0.0001). There was no evidence of publication bias based on the funnel plot (Figure III in the online-only Data Supplement), but 61.6% of the heterogeneity between studies could be accounted for by the age of stroke population >70 years, study period (1960–1989, 1990–1994, 1995–1999, 2000–2005, and 2006–2017), country (Europe, North America, and rest of the world) and ethnicity (white, Afro-Caribbean, and multiracial; Table III in the online-only Data Supplement). Prevalence of prior AF among incident stroke increased from the 1960s onwards before stabilizing around 2000 (Figure 1). The pooled rate for prior AF was higher in Eastern European (22.9%; 19.9–26.0), compared with Western European (18.8%; 16.5–21.1) or non-European studies (16.8%; 13.2–20.3; Figure IVA to IVC in the online-only Data Supplement).

Findings were similar when limited to studies reporting prior AF rates in incident ischemic stroke, with a pooled rate of 17.0% (15.1–18.9) and with the same variables accounting for 70.7% of all heterogeneity between studies (Table IV and Figures V and VI in the online-only Data Supplement). Of the 6 studies that had within-study time trend data on prior AF rates in this group (Figure 1), the Rochester study^36,37^ showed increasing trend from 1960 to 1989, Aosta^28,29^ from 1989 to 2008, Joinville^34^ from 2005 to 2013, and Oxfordshire^8^ from 1981 to 2017. Conversely, the ERMANCIA (1998–2004)^24,25^ and Dijon (1985–2006)^10^ studies showed a reduction in prior AF rates.

**Figure 2.**
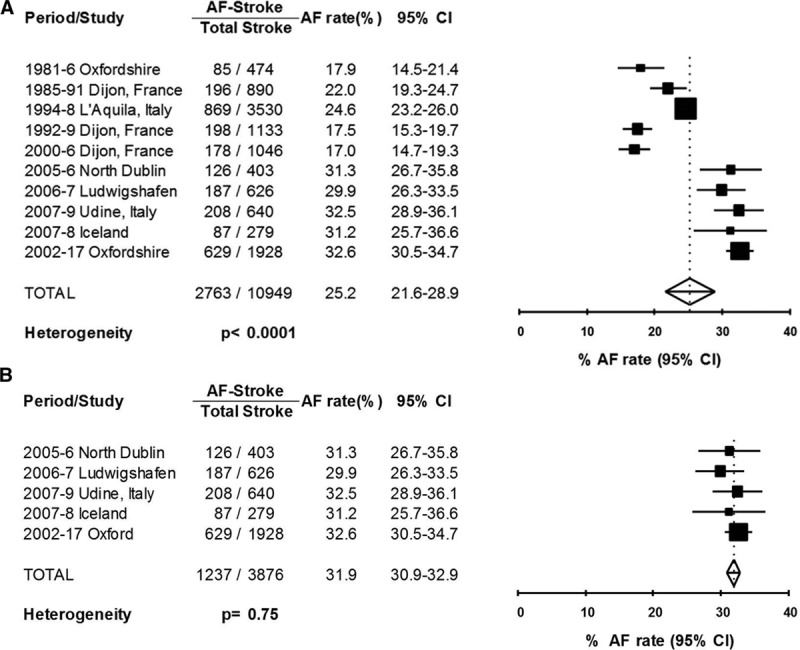
Forest plot of the proportion of incident ischemic stroke associated with total atrial fibrillation (AF; prior and new) across all studies (**A**) and for those completed after 2005 (**B**).

**Figure 3.**
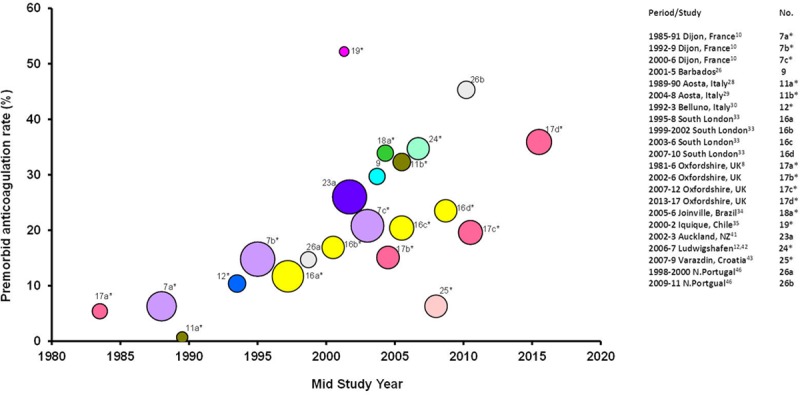
Premorbid anticoagulation in incident ischemic stroke patients with known prior atrial fibrillation (AF). *Incident ischemic stroke.

Of the 8 studies (Figure 2A) that reported the proportion of ischemic stroke associated with any AF, the pooled rate was 25.2% (21.6–28.9, *P*_het_<0.0001), with 97.6% of the heterogeneity between studies accounted for by the above variables. There was a significant increase in pooled rate (*P*=0.001) from 1981 to 2004 (21.6%, 18.3–24.9) compared with after 2005 (Figure 2B: 31.9%, 30.9–32.9; *P*_het_=0.75). The pooled rate of the 4 smaller studies completed after 2005 remained similar (608/1948; 31.2%, 30.0–32.4; *P*_het_=0.75) after excluding the OXVASC data. Of 2746 patients with known prior AF (Table IIA in the online-only Data Supplement), 589 (21.5%, 17.2–25.8) were anticoagulated before stroke onset. Although there has been a significant (*P*=0.002) increase in the pooled rate of premorbid anticoagulation in those with prior AF from before (11.8%, 8.9–14.6) to after 2000 (2001–2015: 25.9% anticoagulated, 21.0–30.9), evidence of substantial undertreatment remained even in the most recent period (Figure 3; ≥2010: 31.6%, 18.2–44.9). In addition, the OXVASC^8^ and Ludwigshafen^12^ studies showed that ≈17% and 36% of strokes with prior AF and CHADS_2_ ≥2 received premorbid anticoagulation, respectively.

## Discussion

To our knowledge, this is the first systematic review of the prevalence of prior AF, total AF, and premorbid anticoagulation in population-based stroke incidence studies. We have shown that 1 in 5 incident strokes had a history of prior AF, rising to 1 in 3 incident ischemic strokes in recent studies, and only about 1 in 5 ischemic stroke patients with prior AF were on premorbid anticoagulation, suggesting substantial undertreatment even after 2010.

Heterogeneity between studies in premorbid AF prevalence among incident strokes (Figure 1) could be mostly explained by the mean age of stroke patients, study period, country of origin, and ethnicity of the population of the respective studies, with the largest portion of heterogeneity explained by ethnicity. This increasing time trend could represent greater awareness of AF as a stroke risk factor together with improved detection in the community and increased AF prevalence with the aging population. Ongoing surveillance is needed to determine whether the recent apparent stabilization of rising prior AF rates is sustained, particularly given the relatively poor long-term compliance with anticoagulation.^48^

Of the 6 studies (Figure V in the online-only Data Supplement) that reported within-study time trends of prior AF prevalence in incident ischemic stroke, 4 reported an increasing prevalence^8,28,29,34,36,37^ and 2^10,24,25^ showed the opposite. The increase in prior AF prevalence in Rochester^36,37^ from 1960s to 1989 could be because of a combination of an aging population and lack of randomized controlled trial evidence for stroke prevention during that era. Rates of premorbid anticoagulation in OXVASC patients with ischemic stroke and prior AF were lowest among the 8 studies completed before 2010 but increased thereafter (Figure 3), possibly because of improved implementation of AF guidelines and increased usage of the direct-acting oral anticoagulants.

The difference between studies in rate of total AF in incident stroke could be partly related to differences in definition of AF-associated stroke and varying rates of cardiac investigations completed to detect AF shortly after onset of stroke. For example, the L’Aquila study^9^ did not include AF occurring within 1 month as AF-associated stroke, whereas the OXVASC,^8^ North Dublin,^11,45^ and Dijon^10^ did. The North Dublin study^11^ had the highest rate of cardiac investigations but did not have significantly higher AF rate than OXVASC^8^ or Ludwigshafen.^12,42^ It is also noteworthy that the different results between OXVASC and Dijon studies occurred despite similar demographics and study periods.^8,10^ The crude reduction of 22.7% in total AF-associated incident ischemic stroke >22 years in Dijon with only modest increase in premorbid anticoagulation (6.3%–21.6%) may have reflected improved management of other vascular risk factors in AF patients and of heart failure in the population.^10^

In a systematic review on oral anticoagulant use in high-risk AF patients in smaller cohort studies,^49^ most studies showed underuse of warfarin with an average rate of 53% (range 16%–96%). Patients who presented to medical attention with ischemic stroke, prior AF, and on anticoagulation belonged to a subset of anticoagulated AF patients in the community. They could reflect an anticoagulation failure in AF patients, subtherapeutic anticoagulation or an alternative stroke cause (eg, concurrent symptomatic carotid stenosis or cancer) other than cardioembolism. The pooled rate for premorbid anticoagulation among patients with incident ischemic stroke and prior AF based on 9 studies (Figure 3) in our systematic review is, therefore, unsurprisingly lower than the above community AF anticoagulation rate.

There are several limitations to our review. First, only 53% of existing population-based stroke incidence studies had reported premorbid or total AF rates among incident ischemic strokes. However, there was a clear trend of increasing incident ischemic stroke associated with prior AF from 1960s to 2000 before stabilizing thereafter. In addition, data from the majority of the studies completed after 2000 were included in our systematic review. Therefore, we think that our results are probably generalizable. Second, with most studies not stating the definition of AF-associated stroke, one could not be certain if atrial flutter was truly included into the AF group, the exclusion of which would lead to under-ascertainment of cases. There was also insufficient reporting of the rate of premorbid anticoagulation, use of preventative medication and the completion of prolonged cardiac monitoring. In addition, standardization of the definition of AF-associated stroke with regards to the time limit of inclusion of new AF detected via prolonged cardiac monitoring would also facilitate comparability of future studies. Future individual patient data meta-analyses might help to examine more closely the inter-relationship of risk factor control and anticoagulation use in AF-associated stroke. Third, there were no studies that could be included from Asia. Forth, as we do not have the data on the primary prevention of AF patients in each population, we cannot reliably estimate the rate of underuse of anticoagulation in the community. Instead, we aim to measure the consequence of underuse of anticoagulation in these population-based studies, thereby highlighting the continued effort needed worldwide to reduce this type of potentially preventable stroke.

In conclusion, about one-third of incident ischemic strokes were associated with AF in recent studies, with evidence of probably substantial underuse of premorbid oral anticoagulation among ischemic strokes with known prior AF, which represents a major opportunity to reduce the burden of stroke at the population level.

## Acknowledgments

We are grateful to all the staff in the general practices that collaborated in OXVASC (Oxford Vascular Study): Abingdon Surgery, Stert St, Abingdon; Malthouse Surgery, Abingdon; Marcham Road Family Health Centre, Abingdon; The Health Centre, Berinsfield; Key Medical Practice; Kidlington; Yarnton Health Centre, Kidlington; 19 Beaumont St, Oxford; East Oxford Health Centre, Oxford; and Church Street Practice, Wantage. We also acknowledge the use of the facilities of the Acute Vascular Imaging Centre, Oxford.

## Sources of Funding

The Oxford Vascular Study is funded by the National Institute for Health Research (NIHR) Oxford Biomedical Research Centre (BRC), Wellcome Trust, Wolfson Foundation and the British Heart Foundation. The views expressed are those of the author(s) and not necessarily those of the NHS, the NIHR or the Department of Health. Dr Béjot received honoraria for or consulting fees from AstraZeneca, Daiichi-Sankyo, BMS, Bayer, Pfizer, Medtronic, MSD, Amgen, and Boehringer-Ingelheim.

## Disclosures

All authors had access to the data and took responsibility for the decision to submit the article.

## Supplementary Material
