# Supplementary material for: Time Trends in Atrial Fibrillation-Associated Stroke and Premorbid Anticoagulation: Population-Based Study and Systematic Review
Source: Stroke. 2018 Nov 29;50(1):21–7. doi: 10.1161/STROKEAHA.118.022249 (PMC6314508; doi:10.1161/STROKEAHA.118.022249)
Supplement: Supplementary file 1 [file str-50-21-s001.pdf]

SUPPLEMENTAL MATERIAL

TIME-TRENDS IN AF-ASSOCIATED STROKE AND PREMORBID ANTICOAGULATION: POPULATION-BASED STUDY AND SYSTEMATIC REVIEW

Gabriel SC Yiin DPhil**, Linxin Li** DPhil, **Yannick Bejot** PhD, **Peter M. Rothwell** FMedSci

Table	title	Page
I	Search terms	2
IIa	Characteristics of population-based studies reporting proportion of incident stroke associated with prior AF	3
IIb	Characteristics of population-based studies reporting proportion of incident stroke or incident ischaemic stroke associated with total AF (prior and new)	6
III	Linear regression determining the degree of heterogeneity attributed to age of stroke population, study period, study country, and ethnicity against AF rate in incident stroke	7
IV	Linear regression determining the degree of heterogeneity attributed to age of stroke population, study period, study country, and ethnicity against AF rate in incident ischaemic stroke	8

Figure	title	Page
I	Projected number of adults individuals with AF in US by 2050 and European Union by 2060	9
II	Flow chart showing selection of studies for systematic review	10
III	Funnel plot of 44 AF rates from 33 reports	11
IVa-c	Forest plot of premorbid AF rates in European and non-European studies	12
V	Premorbid AF prevalence in population-based studies of incident ischaemic stroke	14
VI	Forest plot of premorbid AF rates in studies with incident ischaemic stroke as denominator	15

Table I. Search terms for systematic review and the number of abstracts identified from the 3 databases

Search terms	Abstracts		
	**Pubmed**	**Embase**	**Cochrane**
Population based stroke incidence studies	1986	5835	32
Population based study AND stroke	582	14715	13
Stroke and incidence study	72	36364	773
TOAST and population based study	5	974	0
TOAST	395	7152	48
AF stroke AND population based study	66	22664	0
AF AND rates	1887	12931	211
Atrial flutter and incidence study	0	36948	1
Atrial flutter and population based study	5	24205	0
AF AND stroke incidence	65	5698	5
AF rate AND stroke incidence	0	5496	1
AF rate and population based study	0	10905	132
Population based study AND stroke incidence	43	5487	2
A-S-C-O	3	518	0
Cardioembolic stroke AND population based study	487	1928	25
**Total**	5596	191820	1243

Table IIa. Characteristics of population-based studies reporting proportion of incident stroke associated with known prior AF

Region	Period	Duration (yrs)	Popn	Person-yrs	Ethnicity	Total events	Mean age (SD)	AF rate (%)	OAC rate (%)	AF defn	ECG rate (%)
Tartu, Estonia[1]	1991-1993	3	110631	331893	W	829	69.5 (13.8)	205/829 (24.7)	NR	No	NR
Tartu, Estonia[2]	2001-2003	3	101122	303366	W	451	71.6 (12.3)	135/451 (29.9)	NR	No	NR
Novosibrirsk, Russian[3]	1992-1993	1	158234	158234	W	366	64.8 (13.1)	57/366 (15.6)	NR	No	NR
Kaunas, Lithuania[4]	2004-2005	1	365191	365191	W	803	69.9 (12.8)	204/803 (25.4)	NR	No	NR
Menorca, Spain[4]	2004-2006	2	75135	150270	W	76	72.2 (14.1)	10/76 (13.2)	NR	No	NR
Warsaw, Poland[4]	2005	1	120186	120186	W	132	69 (13.9)	33/132 (25)	NR	No	NR
Grodno, Belarus[5]	2001-2003	3	311134	933402	W	2069	65.8 (11.6)	478/2069 (23.1)	NR	No	NR
Dijon, France*[6]	1985-1991	7	145396	1017772	W	890	NR	143/890 (16.1)	9/143 (6.3)	No	NR
Dijon, France*[6]	1992-1999	8	149759	1198072	W	1133	NR	171/1133 (15.1)	25/171 (14.6)	No	NR
Dijon, France*[6]	2000-2006	7	152415	1066905	W	1046	NR	139/1046 (13.3)	30/139 (21.6)	No	NR
Martinique, French West Indies (ERMANICA)[7]	1998-1999	1	381364	381364	BC	463	71.2 (14.0)	70/463 (15.1)	NR	No	NR
Martinique, French West Indies (ERMANICA)[8]	2003-2004	1	381364	381364	BC	240	73 (11.0)	28/240 (11.7)	NR	No	NR
Barbados[9]	2001-2005	3.5	268762	940667	BC	968	70.8 (15.7)	65/968 (6.7)	19/64 (29.7)	No	NR
Malmo, Sweden[10]	1989-1990	1	232448	232448	W	524	74.8 (NR)	100/524 (19.1)	NR	No	NR
Valley of Aosta, Italy*[11]	1989	1	123748	123748	W	170	72.6 (NR)	31/170 (18.2)	0/31 (0)	No	NR

Valley of Aosta, Italy*[12]	2004-2008	5	125366	626830	W	1057	75.7 (12.7)	235/1057 (22.2)	76/235 (32.3)	N	99%
Belluno, Italy*[13]	1992-1993	1	211389	211389	W	319	NR	77/319 (24.1)	8/77 (10.4)	No	NR
Sesto Fiorentino, Italy[4]	2004-2006	2	47326	94652	W	161	75.9 (11.9)	30/161 (18.6)	NR	No	NR
Aeolian Island, Italy[14]	1999-2002	3	13431	40293	W	62	72.5 (9.9)	7/62 (11.3)	NR	No	NR
Arcardia, Greece[15]	1993-1995	2	80774	161548	W	555	75.4 (NR)	189/555 (34.1)	NR	No	NR
South London,UK[16]	1995-1998	4	234533	938132	MR	1305	NR	252/1223 (20.6)	30/245 (12.2)	No	NR
South London, UK[16]	1999-2002	4	310028	1240112	MR	1074	NR	138/992 (13.9)	21/124 (16.9)	No	NR
South London, UK[16]	2003-2006	4	310028	1240112	MR	994	NR	148/967 (15.3)	30/147 (20.4)	No	NR
South London, UK[16]	2007-2010	4	357308	1429232	MR	877	NR	127/852 (14.9)	29/125 (23.2)	No	NR
Oxfordshire, UK*[17]	1981-1984	4	86517	346068	W	474	77.5 (8.8)	56/474 (11.8)	5/56 (8.9)	Yes	95.3
Oxfordshire, UK*	2002-2017	15	92728	1390920	W	1928	74.5 (13.5)	425/1928 (22.0)	102/425 (24.0)	Yes	97.8
Joinville, Brazil*[18]	2005-2006	2	487047	974094	H	610	66 (13.5)	66/610 (10.8)	22/66 (33.3)	No	NR
Joinville, Brazil*[18]	2012-2013	2	516288	1032576	H	786	64 (15)	109/786 (13.9)	NR	No	NR
Iquique, Chile*[19]	2000-2002	2	214526	429052	H	186	66.5 (14.9)	23/186 (12.4)	12/23 (52.2)	No	76
Rochester, USA*[20,21]	1960-1969	10	NR	NR	W	518	70.4 (NR)	57/518 (11.0)	NR	No	NR
Rochester, USA*[20]	1970-1979	10	52534	525340	W	547	71.0 (NR)	93/547 (17.0)	NR	No	NR

Rochester, USA*[20,21]	1980-1989	10	66596	665960	W	805	73.5 (NR)	177/805 (22.0)	NR	No	NR
Greater Cincinnati, USA*[22,23]	1993-1994	1	1299901	1299901	B&W	1956	71.2 (13.5)	263/1956 (13.4)	NR	No	NR
North Manhattan, USA*[24]	1993-1997	4	210000	840000	MR	695	70.5 (14.0)	132/695 (19.0)	NR	No	NR
Auckland, New Zealand[25]	2002-2003	1	897882	897882	MR	1401	71.8 (14.3)	289/1401 (20.6)	71/289 (24.6)	No	98
Auckland, New Zealand[25]	2011-12	1	1119192	1119192	MR	1329	71.1 (15.2)	389/1329 (29.3)	NR	NR	NR
Ludwigshafen, Germany*[26,27]	2006-2007	2	167906	335812	W	626	71.7 (13.2)	121/626 (19.3)	42/121 (34.7)	Yes	100
Varaždin, Croatia*[28]	2007-2009	2	184115	368230	W	748	72.8 (11.0)	126/748 (16.8)	8/126 (6.3)	No	99.4
Porto, Portugal[29]	1998-2000	2	123112	246224	W	462	72 (NR)	64/462 (13.9)	9/64 (14.1)	NR	NR
Porto, Portugal[29]	2009-2011	2	204444	408888	W	405	78 (NR)	75/405 (18.5)	34/75 (45.3)	NR	NR
Iceland*[30]	2007-2008	1	319355	319355	W	343	72 (NR)	50/279 (17.9)	NR	NR	NR

*Incident ischaemic stroke; Popn=population; W=White; BC=Black Caribbean; H=Hispanics, MR=multiracial, B=black; Defn=definition; OAC=oral anticoagulation; NR=not recorded.

Table IIb. Characteristics of population-based studies reporting proportion of incident stroke or incident ischaemic stroke associated with total AF (prior and new)

Region	Period	Duration (yrs)	Popn	Person-yrs	Ethnicity	Total events	Mean age (SD)	AF rate (%)	OAC rate (%)	AF defn	ECG rate(%)
Oxfordshire, UK*[17]	1981-6	4	86517	346068	W	474	77.3 (9.0)	85/474 (17.9)	3/56 (5.4)	Yes	95.3
Dijon, France*[6]	1985-91	7	145396	1017772	W	890	NR	196/890 (22)	9/143 (6.3)	Yes	NR
L'Aquila, Italy*[31]	1994-8	5	297838	1489190	W	3594	78.8 (13.3)	869/3530 (24.6)	NR	Yes	91.2
Dijon, France*[6]	1992-9	8	149759	1198072	W	1133	NR	198/1133 (17.5)	25/171 (14.6)	Yes	NR
Dijon, France*[6]	2000-6	7	152415	1066905	W	1046	NR	178/1046 (17)	30/139 (21.6)	Yes	NR
North Dublin, UK[32,33]	2005-6	1	294529	294529	W	485	70.2 (14.1)	126/403 (31.1)	NR	Yes	91.6
Ludwigshafen, Germany*[26,27]	2006-7	2	167906	335812	W	626	78 (10)	187/626 (29.9)	42/121 (34.7)	Yes	100
Udine Italy[34]	2007-9	2	153312	306642	W	640	76.2 (13.8)	208/640 (32.5)	NR	Yes	NR
Iceland*[30]	2007-8	1	319355	319355	W	279	72 (NR)	87/279 (31.2)	NR	NR	NR
Oxfordshire, UK*	2002-17	15	92728	1390920	W	1928	74.5 (13.5)	629/1928 (32.6)	102/425 (24.0)	Yes	97.8

*Incident ischaemic stroke; Popn=population; W=White; BC=Black Caribbean; H=Hispanics, MR=multiracial, B=black; Defn=definition; NR=not recorded

Table III. Inverse variance linear regression determining the degree of heterogeneity attributed to age of stroke population, study period, study country and ethnicity against AF rate in incident stroke.

Model Summary

Model	R	R Square	Adjusted R Square	Std. Error of the Estimate	Change Statistics				
					R Square Change	F Change	df1	df2	Sig. F Change
1	.785[a]	.616	.463	317.21149	.616	4.016	10	25	.002

a. Predictors: (Constant), Multiracial, 2006-17, OtherCountries, Age70, 1995-99, 1960-89, 1990-94, Black, NAmerica, White

Coefficients[a,b]

Model		Unstandardized Coefficients		Standardized Coefficients	t	Sig.	95.0% Confidence Interval for B	
		B	Std. Error	Beta			Lower Bound	Upper Bound
1	(Constant)	2.618	5.116		.512	.613	-7.919	13.155
	1960-89	-3.577	3.331	-.197	-1.074	.293	-10.437	3.284
	1990-94	.782	3.090	.046	.253	.802	-5.582	7.146
	1995-99	2.539	3.003	.141	.845	.406	-3.646	8.723
	2006-17	.661	2.225	.047	.297	.769	-3.922	5.244
	Age70	-2.100	2.466	-.151	-.852	.403	-7.178	2.979
	NAmerica	-.677	3.156	-.044	-.215	.832	-7.177	5.823
	OtherCountries	9.458	4.052	.705	2.334	.028	1.113	17.803
	White	19.448	5.481	1.605	3.548	.002	8.159	30.738
	Black	-1.733	4.361	-.093	-.398	.694	-10.714	7.248
	Multiracial	14.073	4.446	1.046	3.166	.004	4.917	23.229

a. Dependent Variable: AFrate_percentage

b. Weighted Least Squares Regression - Weighted by invvariance

Table IV. Inverse variance linear regression determining the degree of heterogeneity attributed to age of stroke population, study period, study country and ethnicity against AF rate in incident ischaemic stroke.

Model Summary

Model	R	R Square	Adjusted R Square	Std. Error of the Estimate	Change Statistics				
					R Square Change	F Change	df1	df2	Sig. F Change
1	.841[a]	.707	.373	250.55570	.707	2.113	8	7	.170

a. Predictors: (Constant), Multiracial, 1995-99, Age70, 2006-17, Black, 2000-05, Europe, 1990-94

Coefficients[a,b]

Model		Unstandardized Coefficients		Standardized Coefficients	t	Sig.	95.0% Confidence Interval for B	
		B	Std. Error	Beta			Lower Bound	Upper Bound
1	(Constant)	4.554	4.308		1.057	.326	-5.632	14.740
	1990-94	.357	6.442	.034	.055	.957	-14.877	15.590
	1995-99	5.904	4.885	.486	1.209	.266	-5.647	17.454
	2000-05	7.216	4.159	.680	1.735	.126	-2.618	17.051
	2006-17	8.556	3.823	.892	2.238	.060	-.484	17.595
	Age70	11.849	4.805	1.065	2.466	.043	.487	23.211
	Europe	-3.303	3.948	-.379	-.837	.430	-12.639	6.032
	Black	-9.055	5.719	-.549	-1.583	.157	-22.577	4.468
	Multiracial	-3.314	6.504	-.343	-.510	.626	-18.693	12.065

a. Dependent Variable: AFrate_percentage

b. Weighted Least Squares Regression - Weighted by invvariance

Figure I. Projected number of adults individuals with AF in US by 2050 and European Union by 2060, assuming stable age-adjusted incidence rates

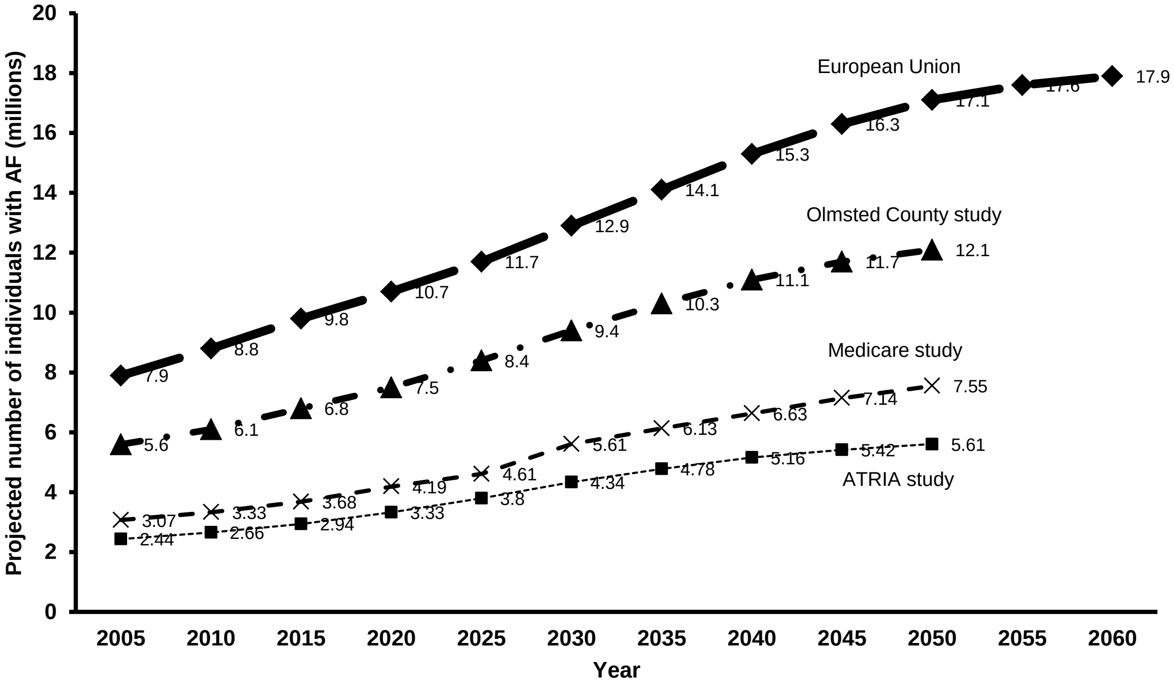

Data derived from ATRIA,[35] Olmsted County,[36] Medicare,[37] and European Union studies[38]

Figure II. Flow chart showing selection of studies for systematic review.

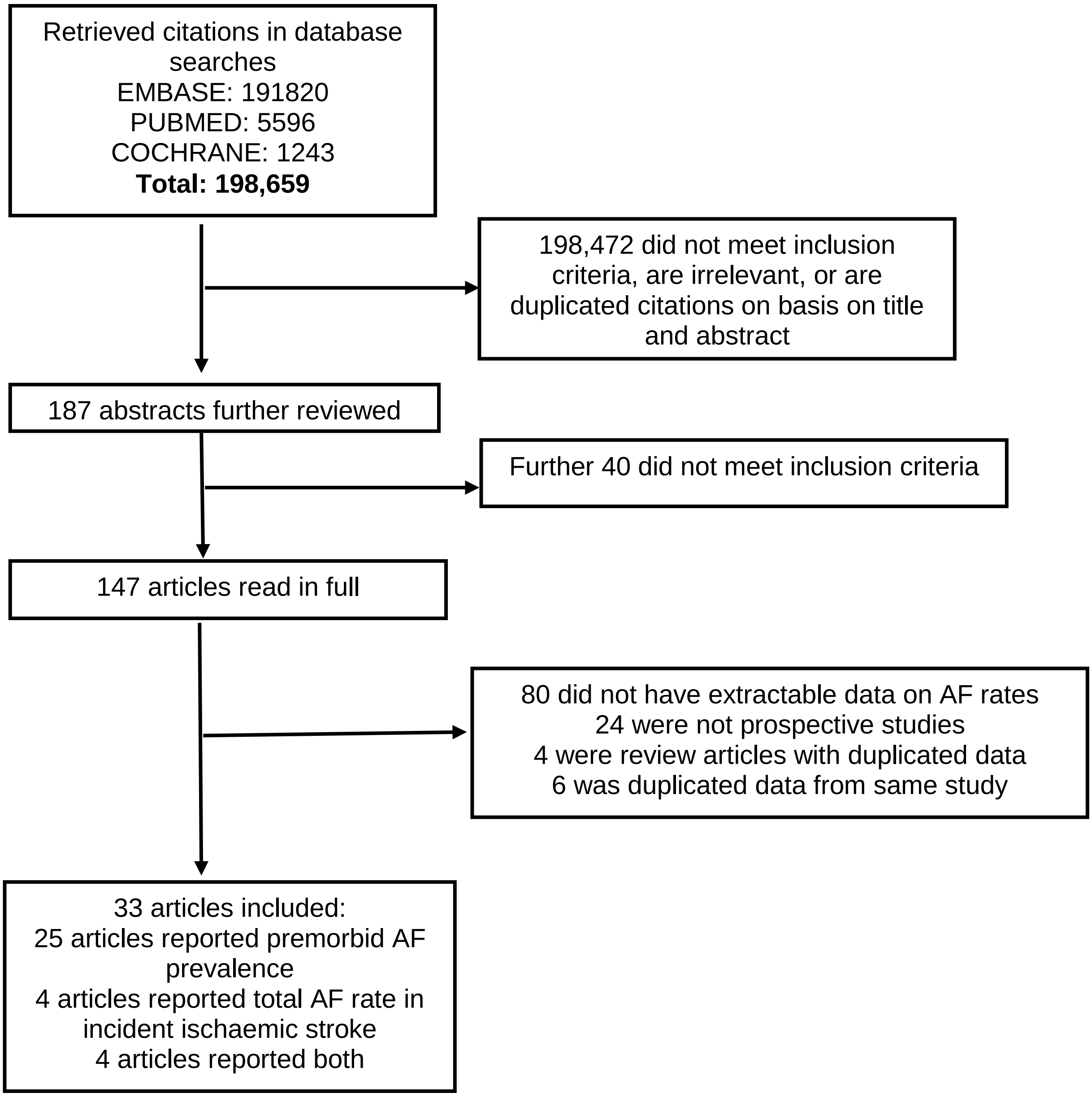

Figure III. Funnel plot of 44 AF rates from 33 reports; SE=standard error

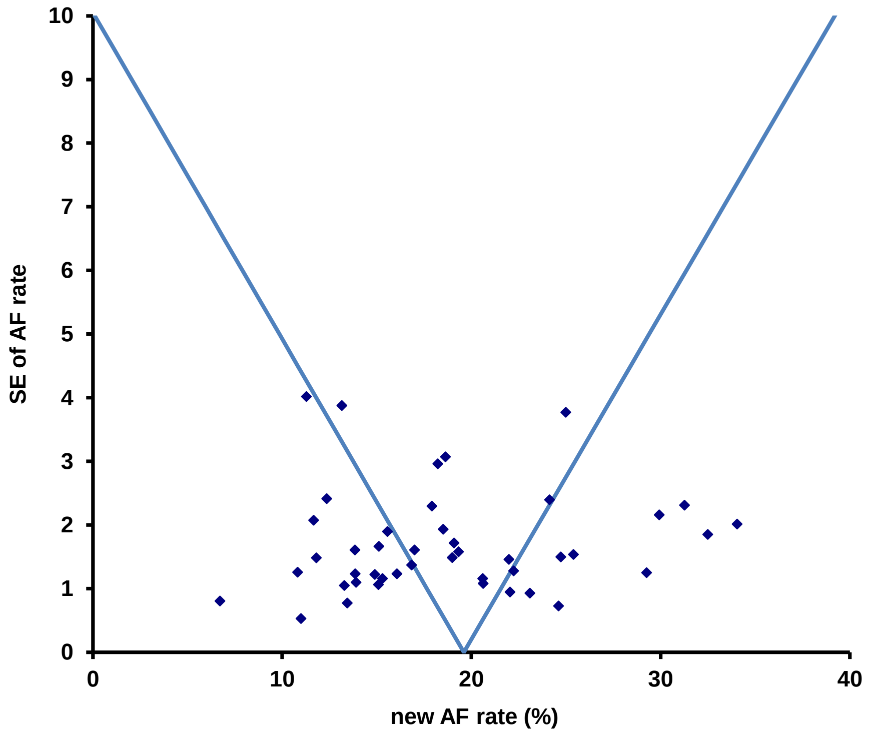

Figure IVa-c. Forest plot of premorbid AF rates in European and non-European studies

(a) Eastern Europe

Period/Study	PreAF / Total stroke	% PreAF	95% CI
1991-3 Tartu, Estonia	205 / 829	24.7	21.8-27.7
1992-3 Novosibrirsk, Russia	57 / 366	15.6	11.9-19.3
2001-3 Grodno, Belarus	478 / 2069	23.1	21.3-24.9
2001-3 Tartu, Estonia	135 / 451	29.9	25.7-34.2
2004-5 Kaunas, Lithuania	204 / 803	25.4	22.4-28.4
2005 Warsaw, Poland	33 / 132	25.0	17.6-32.4
2007-9 Varaždin, Croatia*	126 / 748	16.8	14.2-19.5
TOTAL	1238 / 5398	22.9	19.9-26.0
**Heterogeneity**	**p< 0.0001**		

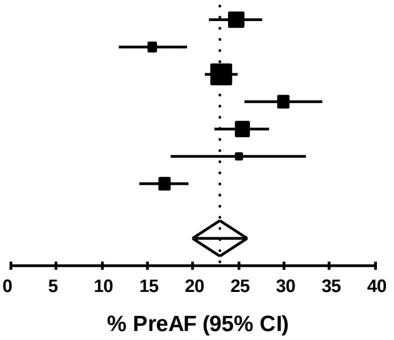

67.1% of the heterogeneity can be accounted for by age of stroke population>70 years, study period (main determinant at 52.3%), and ethnicity of population

(b) Western Europe

Period/Study	PreAF / Total stroke	% PreAF	95% CI
1981-6 Oxfordshire, UK*	56 / 474	11.8	8.9-14.7
1985-91 Dijon, France*	143 / 890	16.1	13.7-18.5
1989-90 Malmo, Sweden	100 / 524	19.1	15.7-22.4
1989-90 Valley of Aosta, Italy*	31 / 170	18.2	12.4-24.0
1989-90 Valley of Aosta, Italy*	235 / 1057	22.2	19.7-24.7
1992-3 Belluno, Italy*	77 / 319	24.1	19.4-28.8
1993-5 Arcardia, Greece	189 / 555	34.1	30.1-38.0
1995-8 South London, UK	252 / 1223	20.6	18.3-22.9
1992-9 Dijon, France*	171 / 1133	15.1	13.0-17.2
1999-2002 South London, UK	138 / 992	13.9	11.8-16.1
1998-2000 N.Portugal	64 / 462	13.9	10.7-17.0
2003-6 South London, UK	148 / 967	15.3	13.0-17.6
1999-2002 Aeolian Island	7 / 62	11.3	3.4-19.2
2007-10 South London, UK	127 / 852	14.9	12.5-17.3
2000-6 Dijon, France*	139 / 1046	13.3	11.2-15.3
2004-6 Menorca, Spain	10 / 76	13.2	5.6-20.8
2004-6 Sesto Fiorentino, Italy	30 / 161	18.6	12.6-24.6
2007-9 Udine, Italy	208 / 640	32.5	28.9-36.1
2006-7 Ludwigshafen, Germany*	121 / 626	19.3	16.2-22.4
2009-11 N.Portugal	75 / 405	18.5	14.7-22.3
2007-8 Iceland	50 / 279	17.9	13.4-22.4
2002-2017 Oxfordshire, UK*	425 / 1928	22.0	20.2-23.9
TOTAL	2796 / 14841	18.8	16.5-21.1
**Heterogeneity**	**p= 0.0000**		

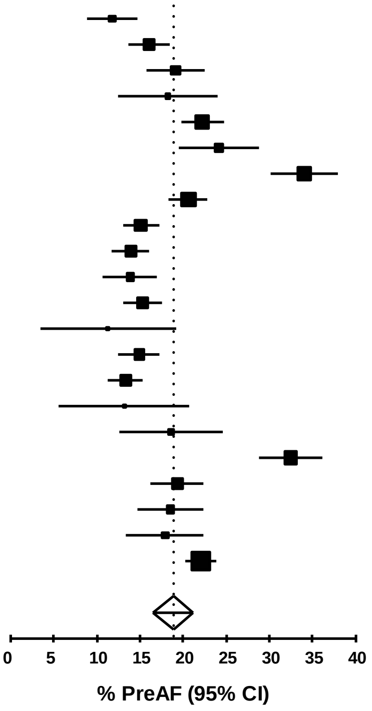

76.5% of the heterogeneity can be accounted for by age of stroke population>70 years, study period (main determinant at 48.2%) and ethnicity of population

(c) Rest of the world

Period/Study	PreAF / Total stroke	% PreAF	95% CI
1960-9 Rochester, USA*	57 / 518	11.0	8.3-13.7
1970-9 Rochester, USA*	93 / 547	17.0	13.9-20.1
1980-9 Rochester, USA*	177 / 805	22.0	19.1-24.8
1993-4 GCNK, USA*	263 / 1956	13.4	11.9-15.0
1993-7 N.Manhattan, USA*	132 / 695	19.0	16.1-21.9
1998-9 French West Indies*	70 / 463	15.1	11.9-18.4
2000-2 Iquique, Chile*	23 / 186	12.4	7.6-17.1
2001-05 Barbados	65 / 968	6.7	5.1-8.3
2002-3 Auckland, NZ	289 / 1401	20.6	18.5-22.7
2011-12 Auckland, NZ	389 / 1329	29.3	26.8-31.7
2003-4 French West Indies*	28 / 240	11.7	7.6-15.7
2005-6 Joinville, Brazil*	66 / 610	10.8	8.4-13.3
2012-13 Joinville, Brazil*	109 / 786	13.9	11.5-16.3
TOTAL	1761 / 10504	16.8	13.2-20.3
**Heterogeneity**	**p< 0.0001**		

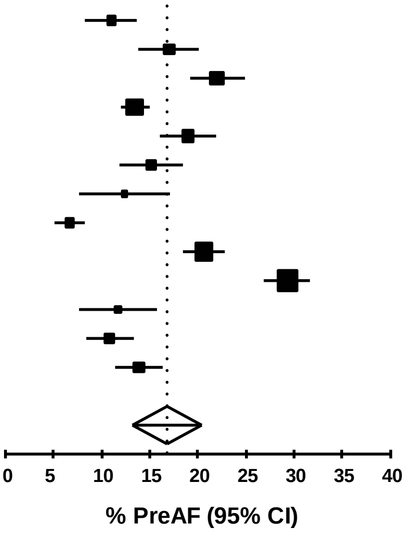

81.6% of the heterogeneity can be accounted for by age of stroke population>70 years, study period and ethnicity (main determinant at 45.1%) of population

PreAF indicates premorbid AF

Figure V. Premorbid AF prevalence in population-based studies of incident ischaemic stroke*

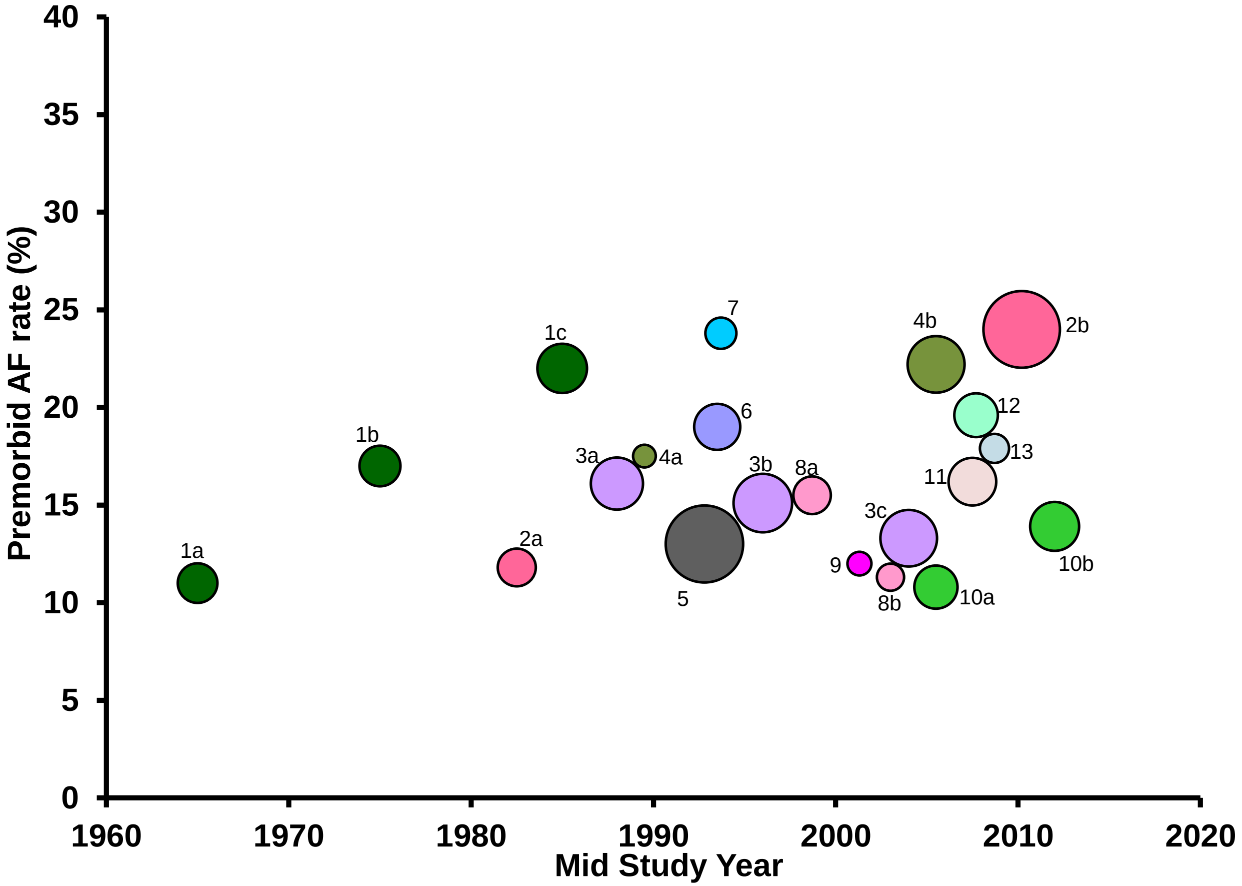

Study	No.
1960-9 Rochester, USA[20,21]	1a
1970-9 Rochester, USA[20]	1b
1980-9 Rochester, USA[20,21]	1c
1981-6 Oxford, UK[17]	2a
2002-17 Oxford, UK	2b
1985-91 Dijon, France[6]	3a
1992-9 Dijon, France[6]	3b
2000-6 Dijon, France[6]	3c
1989-90 Aosta, Italy[11]	4a
2004-8 Aosta, Italy[12]	4b
1993-4 GCNK, USA[22,23]	5
1993-7 North Manhattan[24]	6
1992-3 Belluno, Italy[13]	7
1998-9 French West Indies[7]	8a
2003-4 French West Indies[8]	8b
2000-2 Iquique, Chile[19]	9
2005-6 Joinville, Brazil[18]	10a
2012-13 Joinville, Brazil[18]	10b
2007-9 Varaždin, Croatia[28]	11
2006-7 Ludwigshafen[26,27]	12
2007-8 Iceland[30]	13

Overall heterogeneity p<0.0001, of which 70.7% could be accounted for by age of stroke population >70years, study period (main determinant at 36.1%), country of origin and ethnicity of population - see Online-only data supplement Table IV.

Figure VI. Forest plot of premorbid AF rates in studies with incident ischaemic stroke* as denominator

Studies	PreAF / Total screened	% PreAF	95% CI
1960-9 Rochester, USA*	57 / 518	11.0	8.3-13.7
1970-9 Rochester, USA*	93 / 547	17.0	13.9-20.1
1981-6 Oxford, UK*	56 / 474	11.8	8.9-14.7
1980-9 Rochester, USA*	177 / 805	22.0	19.1-24.8
1985-91 Dijon, France*	143 / 890	16.1	13.7-18.5
1989-90 Valley of Aosta, Italy*	31 / 170	18.2	12.4-24.0
1992-3 Belluno, Italy*	77 / 319	24.1	19.4-28.8
1993-4 GCNK, USA*	263 / 1956	13.4	11.9-15.0
1993-7 N.Manhattan, USA*	132 / 695	19.0	16.1-21.9
1992-9 Dijon, France*	171 / 1133	15.1	13.0-17.2
1998-9 French West Indies*	70 / 463	15.1	11.9-18.4
2000-2 Iquique, Chile*	23 / 186	12.4	7.6-17.1
2003-4 French West Indies*	28 / 240	11.7	7.6-15.7
2000-6 Dijon, France*	139 / 1046	13.3	11.2-15.3
2005-6 Joinville, Brazil*	66 / 610	10.8	8.4-13.3
2006-7 Ludwigshafen, Germany*	121 / 626	19.3	16.2-22.4
2004-8 Valley of Aosta, Italy*	235 / 1057	22.2	19.7-24.7
2007-9 Varaždin, Croatia*	126 / 748	16.8	14.2-19.5
2007-8 Iceland*	87 / 279	31.2	25.7-36.6
2012-13 Joinville, Brazil*	109 / 786	13.9	11.5-16.3
2002-17 Oxford, UK*	425 / 1928	22.0	20.2-23.9
TOTAL	2629 / 15476	17.0	15.1-18.9
**Heterogeneity**	**p< 0.0001**		

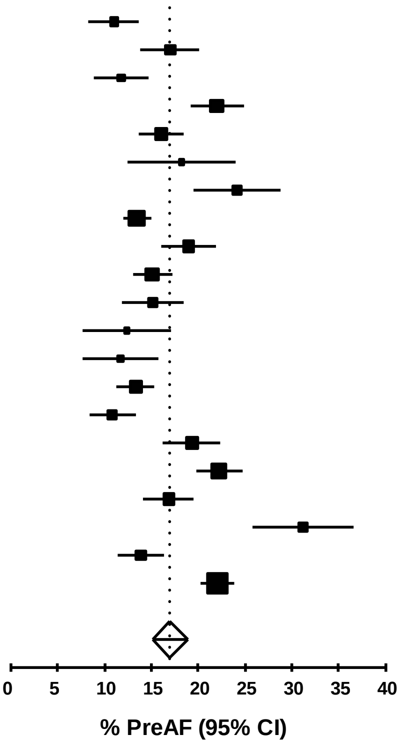

70.7% of the heterogeneity can be accounted for by age of stroke population>70 years, study period and ethnicity (main determinant at 25.5%) of population

Supplemental References

1. Korv J RM, Kaasik A-E. Stroke registry of Tartu, Estonia, from 1991 through 1993. *Cerebrovasc Dis.* 1997;7:154-162
2. Vibo R, Korv J, Roose M. The third stroke registry in Tartu, Estonia: Decline of stroke incidence and 28-day case-fatality rate since 1991. Stroke. 2005;36:2544-2548
3. Feigin VL, Wiebers DO, Nikitin YP, O'Fallon WM, Whisnant JP. Stroke epidemiology in Novosibirsk, Russia: A population-based study. *Mayo Clin Proc.* 1995;70:847-852
4. Heuschmann PU, Di Carlo A, Bejot Y, Rastenyte D, Ryglewicz D, Sarti C, et al. Incidence of stroke in Europe at the beginning of the 21st century. *Stroke.* 2009;40:1557-1563
5. Kulesh SD, Filina NA, Frantava NM, Zhytko NL, Kastsinevich TM, Kliatskova LA, et al. Incidence and case-fatality of stroke on the east border of the European Union: The Grodno stroke study. *Stroke.* 2010;41:2726-2730
6. Bejot Y, Ben Salem D, Osseby GV, Couvreur G, Durier J, Marie C, et al. Epidemiology of ischemic stroke from atrial fibrillation in Dijon, France, from 1985 to 2006. *Neurology.* 2009;72:346-353
7. Smadja D, Cabre P, May F, Fanon JL, Rene-Corail P, Riocreux C, et al. ERMANCIA: Epidemiology of stroke in Martinique, French West Indies: Part i: Methodology, incidence, and 30-day case fatality rate. *Stroke.* 2001;32:2741-2747
8. Chausson N, Olindo S, Cabre P, Saint-Vil M, Smadja D. Five-year outcome of a stroke cohort in Martinique, French West Indies: Etude Realisee en Martinique et Centree sur l'incidence des Accidents vasculaires cerebraux, part 2. *Stroke.* 2010;41:594-599
9. Smeeton NC, Corbin DO, Hennis AJ, Hambleton IR, Fraser HS, Wolfe CD, et al. Differences in risk factors between black caribbean patients with stroke in Barbados and South London. *Stroke.* 2009;40:640-643
10. Jerntorp P, Berglund G. Stroke registry in Malmo, Sweden. Stroke. 1992;23:357-361
11. D'Alessandro G, Di Giovanni M, Roveyaz L, Iannizzi L, Compagnoni MP, Blanc S, et al. Incidence and prognosis of stroke in the Valle D'Aosta, Italy. First-year results of a community-based study. *Stroke.* 1992;23:1712-1715
12. Corso G, Bottacchi E, Giardini G, Di Giovanni M, Meloni T, Campagnoni MP, et al. Epidemiology of stroke in northern Italy: The cerebrovascular Aosta registry, 2004-2008. *Neurological Sciences.* 2013;34:1071-1081
13. Lauria G, Gentile M, Fassetta G, Casetta I, Agnoli F, Andreotta G, et al. Incidence and prognosis of stroke in the Belluno province, Italy. First-year results of a community-based study. *Stroke.* 1995;26:1787-1793
14. Musolino R, La Spina P, Serra S, Postorino P, Calabro S, Savica R, et al. First-ever stroke incidence and 30-day case fatality in the Sicilian Aeolian archipelago, Italy. *Stroke.* 2005;36:2738-2741
15. Vemmos KN, Bots ML, Tsibouris PK, Zis VP, Grobbee DE, Stranjalis GS, et al. Stroke incidence and case fatality in southern Greece: The Arcadia stroke registry. *Stroke.* 1999;30:363-370
16. Marshall IJ, Wang YZ, McKevitt C, Rudd AG, Wolfe CDA. Trends in risk factor prevalence and management before first stroke data from the South London Stroke Register 1995-2011. *Stroke.* 2013;44:1809-1816
17. Yiin GS, Howard DP, Paul NL, Li L, Luengo-Fernandez R, Bull LM, et al. Age-specific incidence, outcome, cost, and projected future burden of atrial fibrillation-related embolic vascular events: A population-based study. *Circulation.* 2014;130:1236-1244
18. Cabral NL, Cougo-Pinto PT, Magalhaes PSC, Longo AL, Moro CHC, Amaral CH, et al. Trends of stroke incidence from 1995 to 2013 in Joinville, Brazil. *Neuroepidemiology.* 2016;46:273-281
19. Lavados PM, Sacks C, Prina L, Escobar A, Tossi C, Araya F, et al. Incidence, 30-day case-fatality rate, and prognosis of stroke in Iquique, Chile: A 2-year community-based prospective study (PISCIS project). *Lancet.* 2005;365:2206-2215
20. Tsang TS, Petty GW, Barnes ME, O'Fallon WM, Bailey KR, Wiebers DO, et al. The prevalence of atrial fibrillation in incident stroke cases and matched population controls in Rochester, Minnesota: Changes over three decades. *J Am Coll Cardiol.* 2003;42:93-100
21. Brown RD, Whisnant JP, Sicks JD, O'Fallon WM, Wiebers DO. Stroke incidence, prevalence, and survival: Secular trends in Rochester, Minnesota, through 1989. *Stroke.* 1996;27:373-380
22. Schneider AT, Kissela B, Woo D, Kleindorfer D, Alwell K, Miller R, et al. Ischemic stroke subtypes: A population-based study of incidence rates among blacks and whites. *Stroke.* 2004;35:1552-1556

23. Kleindorfer DO, Khoury J, Moomaw CJ, Alwell K, Woo D, Flaherty ML, et al. Stroke incidence is decreasing in whites but not in blacks: A population-based estimate of temporal trends in stroke incidence from the Greater Cincinnati/Northern Kentucky stroke study. *Stroke.* 2010;41:1326-1331
24. White H, Boden-Albala B, Wang C, Elkind MS, Rundek T, Wright CB, et al. Ischemic stroke subtype incidence among whites, blacks, and hispanics: The Northern Manhattan study. *Circulation.* 2005;111:1327-1331
25. Krishnamurthi RV, Barker-Collo S, Parag V, Parmar P, Witt E, Jones A, et al. Stroke incidence by major pathological type and ischemic subtypes in the Auckland Regional Community Stroke Studies changes between 2002 and 2011. *Stroke.* 2018;49:3-10
26. Palm F, Kleemann T, Dos Santos M, Urbanek C, Buggle F, Safer A, et al. Stroke due to atrial fibrillation in a population-based stroke registry (Ludwigshafen Stroke Study) CHA(2) , DS(2) -VASc score, underuse of oral anticoagulation, and implications for preventive measures. *Eur J Neurol.* 2012;21:117-123
27. Palm F, Urbanek C, Wolf J, Buggle F, Kleemann T, Hennerici MG, et al. Etiology, risk factors and sex differences in ischemic stroke in the Ludwigshafen stroke study, a population-based stroke registry. *Cerebrovasc Dis.* 2012;33:69-75
28. Pikija S, Cvetko D, Malojcic B, Trkanjec Z, Pavlicek I, Lukic A, et al. A population-based prospective 24-month study of stroke: Incidence and 30-day case-fatality rates of first-ever strokes in Croatia. *Neuroepidemiology.* 2012;38:164-171
29. Correia M, Magalhaes R, Felgueiras R, Quintas C, Guimaraes L, Silva MC. Changes in stroke incidence, outcome, and associated factors in Porto between 1998 and 2011. *International Journal of Stroke.* 2017;12:169-179
30. Hilmarsson A, Kjartansson O, Olafsson E. Incidence of first stroke: a population study in Iceland. *Stroke.* 2013;44:1714-1716
31. Marini C, De Santis F, Sacco S, Russo T, Olivieri L, Totaro R, et al. Contribution of atrial fibrillation to incidence and outcome of ischemic stroke: Results from a population-based study. *Stroke.* 2005;36:1115-1119
32. Hannon N, Sheehan O, Kelly L, Marnane M, Merwick A, Moore A, et al. Stroke associated with atrial fibrillation--incidence and early outcomes in the North Dublin population stroke study. *Cerebrovasc Dis.* 2010;29:43-49
33. Kelly PJ, Crispino G, Sheehan O, Kelly L, Marnane M, Merwick A, et al. Incidence, event rates, and early outcome of stroke in Dublin, Ireland the North Dublin population stroke study. *Stroke.* 2012;43:2042-2047
34. Janes F, Gigli GL, D'Anna L, Cancelli I, Perelli A, Canal G, et al. Stroke incidence and 30-day and six-month case fatality rates in Udine, Italy: A population-based prospective study. *International Journal of Stroke.* 2013;8:100-105
35. Go AS, Hylek EM, Phillips KA, Chang Y, Henault LE, Selby JV, et al. Prevalence of diagnosed atrial fibrillation in adults: national implications for rhythm management and stroke prevention: the AnTicoagulation and Risk Factors in Atrial Fibrillation (ATRIA) Study. *JAMA.* 2001;285:2370-2375
36. Miyasaka Y, Barnes ME, Gersh BJ, Cha SS, Bailey KR, Abhayaratna WP, et al. Secular trends in incidence of atrial fibrillation in Olmsted County, Minnesota, 1980 to 2000, and implications on the projections for future prevalence. *Circulation.* 2006;114:119-125
37. Naccarelli GV, Varker H, Lin J, Schulman KL. Increasing prevalence of atrial fibrillation and flutter in the United States. *Am J Cardiol.* 2009;104:1534-1539
38. Krijthe BP, Kunst A, Benjamin EJ, Lip GY, Franco OH, Hofman A, et al. Projections on the number of individuals with atrial fibrillation in the European Union, from 2000 to 2060. *European Heart J.* 2013;34:2746-2751
